# Economic Evaluation of Apixaban for the Prevention of Stroke in Non-Valvular Atrial Fibrillation in the Netherlands

**DOI:** 10.1371/journal.pone.0103974

**Published:** 2014-08-05

**Authors:** Jelena Stevanović, Marjolein Pompen, Hoa H. Le, Mark H. Rozenbaum, Robert G. Tieleman, Maarten J. Postma

**Affiliations:** 1 University of Groningen, Groningen, the Netherlands; 2 Bristol-Myers Squibb, Woerden, the Netherlands; 3 Pfizer Nederland, Capelle aan den IJssel, the Netherlands; 4 Martini Hospital, Groningen, the Netherlands; University Hospital Medical Centre, Germany

## Abstract

**Background:**

Stroke prevention is the main goal of treating patients with atrial fibrillation (AF). Vitamin-K antagonists (VKAs) present an effective treatment in stroke prevention, however, the risk of bleeding and the requirement for regular coagulation monitoring are limiting their use. Apixaban is a novel oral anticoagulant associated with significantly lower hazard rates for stroke, major bleedings and treatment discontinuations, compared to VKAs.

**Objective:**

To estimate the cost-effectiveness of apixaban compared to VKAs in non-valvular AF patients in the Netherlands.

**Methods:**

Previously published lifetime Markov model using efficacy data from the ARISTOTLE and the AVERROES trial was modified to reflect the use of oral anticoagulants in the Netherlands. Dutch specific costs, baseline population stroke risk and coagulation monitoring levels were incorporated. Univariate, probabilistic sensitivity and scenario analyses on the impact of different coagulation monitoring levels were performed on the incremental cost-effectiveness ratio (ICER).

**Results:**

Treatment with apixaban compared to VKAs resulted in an ICER of €10,576 per quality adjusted life year (QALY). Those findings correspond with lower number of strokes and bleedings associated with the use of apixaban compared to VKAs. Univariate sensitivity analyses revealed model sensitivity to the absolute stroke risk with apixaban and treatment discontinuations risks with apixaban and VKAs. The probability that apixaban is cost-effective at a willingness-to-pay threshold of €20,000/QALY was 68%. Results of the scenario analyses on the impact of different coagulation monitoring levels were quite robust.

**Conclusions:**

In patients with non-valvular AF, apixaban is likely to be a cost-effective alternative to VKAs in the Netherlands.

## Introduction

Atrial fibrillation (AF) is a heart disease common among elderly people. In the Netherlands incidence rates increase with advancing age from approximately 1% among 55-year olds to 18% among 85-year olds and related relevant risks of ischemic stroke (IS) and other systemic thromboembolic events [1], [2]. In addition, patients with AF suffer not only from a greater activity impairment and lower quality of life (QoL) compared to the general population but also have a 50–90% increased risk of mortality [3], [4]. The majority of AF patients suffer from non-valvular AF. Strokes related to AF are often characterized by more severe disability and impairment of QoL in comparison to strokes due to other causes [5]. As a result, stroke related morbidity, which is driven by high hospitalization and long-term maintenance costs, causes a high economic burden to the Dutch health care system. Specifically, the 6-month cost of usual care for stroke patients range from €16,000 to €54,000 depending on severity [6]. In parallel, the annual costs of treating patients with AF in the Netherlands were estimated to mount up to €2,328 with 70.1% of the resources allocated to the inpatient care and interventional procedures [7]. Given the humanistic implications of both AF and stroke and economic considerations of their management, stroke prevention is the main focus of treatment strategies for patients with AF and could be expected to lead to both health and economic benefits.

Until recently patients with AF and an estimated moderate to high risk of stroke (i.e. cardiac failure, hypertension, age, diabetes, stroke (doubled) [CHADS_2_] score ≥2) were recommended to receive vitamin-K antagonists (VKAs; e.g. warfarin, acenocoumarol or phenprocoumon) for stroke prevention [8]. However, although VKAs present a highly effective treatment strategy in reducing the incidence of stroke, their optimal effectiveness and safety is crucially safeguarded with regular coagulation monitoring due to VKAs’ narrow therapeutic range (international normalized ratio [INR] limits of 2.0 and 3.0) [9]. Failure to achieve the anticoagulant effect inside the required INR therapeutic range increases the risk of IS and bleeding including hemorrhagic stroke (HS). The complexity of regular monitoring, which in the Dutch healthcare system is handled by thrombotic services, possibly followed by failure to achieve the safety range inside INR limits, accompanied with multiple drug and food interactions, might lead to underuse of VKAs or even result in an increase in medication-related hospital admissions as observed in HARM study [8], [10].

Recently, a new class of anticoagulants became available (novel oral anticoagulant (NOAC)) that are at least as effective or superior in reducing the risk of stroke or systemic embolism (SE), have a better efficacy/safety profile and exclude the need for constant INR monitoring, compared to VKAs [11]–[13]. Accordingly, NOACs have been included in both international and national guidelines [8], [14]. One of them is apixaban, a NOAC of which the efficacy and safety was tested in clinical trials on VKA suitable (ARISTOTLE trial [ClinicalTrials.gov Identifier, NCT00412984]) or unsuitable (AVERROES trial [ClinicalTrials.gov Identifier, NCT00496769]) non-valvular AF patients with a high risk of stroke [11], [15]. In the AVERROES trial, apixaban was shown to prevent more stroke or SE events with no significant difference in the incidence of major bleedings (MBs) or intracranial hemorrhages (ICHs) compared to acetylsalicylic acid (ASA) [15]. Similarly, in the ARISTOTLE trial, less stroke or SE events, less MBs and less fatal events related to any cause were observed in the treatment with apixaban when compared to the treatment with VKA [11]. Despite obvious advantages of the NOACs, the choice of the optimal treatment strategy for AF-patients always needs to be made with respect to both health and economic consequences of the approach chosen, including a formal comparison of apixaban and VKAs as one element [16].

The aim of this study is to evaluate the health and economic consequences of applying apixaban compared to VKAs for stroke prevention in non-valvular AF patients in the Netherlands. The health consequences associated with the use of apixaban and VKAs reflecting the likelihoods of having stroke, other thromboembolic or bleeding events, are mainly based on the data from the ARISTOTLE trial [11]. The cost estimates of stroke and other AF-related complications as well as drug costs, reflect the Dutch situation from the healthcare payers’ perspective.

## Methods

### Decision model

**Figure 1:**
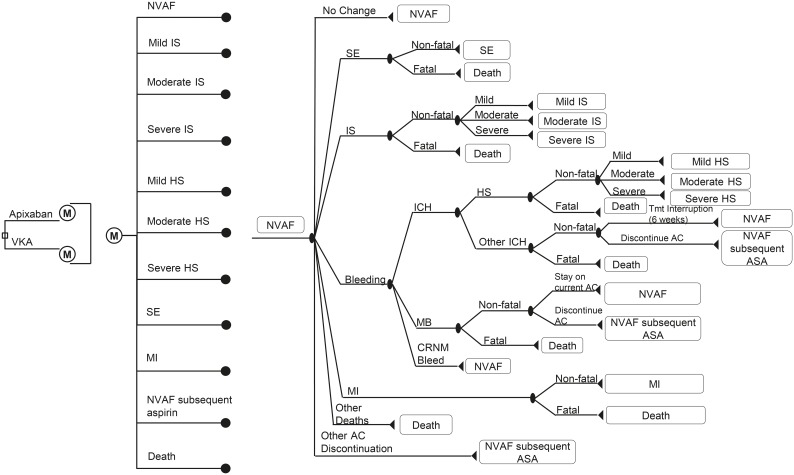
Model for the non-valvular AF population. Depicted in the diagram are the chance nodes (circles) and terminal nodes (triangles). Branches for apixaban and VKA are identical except numerical risks. Patients that discontinue the initial anticoagulant treatment re-enter the model with identical Markov branches but under the assumption of switching their treatment to acetylsalicylic acid. NVAF, non-valvular atrial fibrillation; ASA, acetylsalicylic acid; IS, ischemic stroke; HS, hemorrhagic stroke; SE, systemic embolism; MI, myocardial infarction; ICH, intracranial hemorrhage; CRNM, clinically-relevant non-major; MB, major bleeding; Tmt, treatment.

Previously published lifetime Markov model was modified and updated to reflect the use of apixaban per defined daily dose and adjusted-dose warfarin in patients with non-valvular AF in the Netherlands [17], [18]. The following health states were included in the model: baseline (non-valvular AF), IS, HS, SE, myocardial infarction (MI), other ICH, other MB and clinically-relevant non-major (CRNM) bleeding, other treatment discontinuation and death (Figure 1). Notably, other treatment discontinuations reflect discontinuations that are not directly related to having had a thrombotic or bleeding event. For the purposes of this study, warfarin, studied versus apixaban in the ARISTOTLE trial [11], was used as a comparator, as the Dutch reimbursement authorities presume the efficacy and safety profile of warfarin and acenocoumarol/phenprocoumon (also VKAs) to be interchangeable [19].

Base-case analysis followed a cohort of 1,000 patients with non-valvular AF whose characteristics were comparable to those in the ARISTOTLE trial. Specifically, patients were predominantly male, aged 70 years, with an average CHADS_2_ score of 2.3 and a history of previous VKA use (Table 1) [11], [20], [21]. The progression of patients with non-valvular AF through the Markov model is detailed elsewhere [17], [18]. Briefly, patients remained in the baseline state until a fatal or non-fatal event or treatment discontinuation occurred, or they died due to other, non-cardiovascular related, causes. In order to reflect daily life more closely, a distinction between different levels of IS and HS severity was made in the model, i.e. mild, moderate, severe and fatal. The model allows one recurrent stroke event to occur. The annual risk of recurrent stroke event was based on the 10-year cumulative risk of recurrence derived from a population based study using the South London stroke registry [22]. Health states for thromboembolic events other than stroke (i.e. SE and MI) were considered to be absorbing (i.e. patients remain there until death). The probability of patient being in a particular health state was assessed every 6-weeks which was the cycle length of the model.

**Table 1:** Baseline characteristics of the patients included in the model.

Characteristic	Value	Range	Reference
Age	70	63–77	[11]
Gender (female, %)	35.3	34.1–36.5	[11]
CHADS_2_ (% of patients)			
1	7	5.4–8.8	[20]
2	27	21.3–33.1	[20]
3	25	19.7–30.7	[20]
4	20	15.7–24.7	[20]
5	12	9.3–15	[20]
6	7	5.4–8.8	[20]
7	2	1.5–2.5	[20]
Average TTR in the Netherlands (%)	72.48	Fixed	[21]

CHADS_2_, cardiac failure, hypertension, age, diabetes, stroke (doubled); TTR, time in therapeutic range.

Certain assumptions on the treatment following thromboembolic or bleeding events were made. Firstly, upon the occurrence of IS or SE, patients surviving were assumed to stay on the initially assigned anticoagulant treatment while those surviving HS and MI were assumed to only to receive long-term disease-specific maintenance treatment. Secondly, patients experiencing other ICH, MB and CRNM bleeding were allocated between an option to stay on the initially assigned treatment and an option to switch to ASA. Details on the allocation of patients between the two treatment options are provided in previously published studies [17], [18]. Patients staying on the initially assigned anticoagulant treatment after an ICH that was not a HS, were additionally assumed to have a six-week drug holiday. Finally, patients discontinuing the initial treatment for reasons unrelated to stroke, SE and bleeding were assumed to switch to ASA.

The final outcome of the decision model is the incremental cost-effectiveness ratio (ICER) of apixaban compared to VKA. As a measure of effectiveness, quality-adjusted life-years (QALYs) and life years (LYs) gained were estimated. All relevant costs incorporated in the model reflect the health care payer’s perspective and were inflated to price year 2013 using the Dutch consumer price index [23]. Future costs and health effects were discounted by 4% and 1.5% annually after the first year, according to the Dutch guidelines for pharmacoeconomic research [24].

### Transition probabilities

Data from the ARISTOTLE and the AVERROES trial were the main sources used to estimate the transition probabilities between the health states in the model for patients receiving apixaban, VKA and ASA [11], [15], [17], [18]. Specifically, the rates of IS, MI, SE, ICH, other MB and CRNM bleeding and other treatment discontinuations from the aforementioned trials, were applied for deriving the transition probabilities between the health states similarly to previously published Markov models (Table S1) [17], [18]. Additionally, trial rates of IS, ICH, other MB and CRNM bleeding were adjusted for the average level of risk dependent on the level of INR control in the Netherlands represented by mean time in therapeutic range (TTR) (i.e. 72.48% [21], [25]). ICHs were further differentiated into HSs and other ICHs; other MBs were differentiated to those that were or were not gastrointestinal (GI) by location. Details on the number of patients experiencing one of the two types of ICHs, specific fatality rates after stroke, MI, SE, ICH and other MB and the factors of age-related increasing risk of stroke, bleeding (i.e. ICH, other MB and CRNM bleedings) and MI are provided elsewhere [17], [18], [26].

The average risk of IS in patients receiving apixaban and VKA, was estimated as the joint probability of having an event associated with a specific baseline population stroke risk represented by CHADS_2_ score corrected for the average level of risk dependent on INR control, and the probability of having an event associated with the level of INR control in the Netherlands (Table 1) [21], [25]. Baseline population stroke risk represented by CHADS_2_ score was determined by weighting the risk for each categorization of CHADS_2_ score by the proportion of patients within each group of CHADS_2_ score in the Netherlands [20].

Published population based registries were used to estimate the transition probabilities for recurrence of events [22]. The annual risks for recurrent stroke events of 2.97 and 2.17 were assigned to patients surviving first IS and HS, respectively [22]. The distribution of stroke severity for recurrent stroke events was assumed to be the same as that of the first stroke events in patients treated with apixaban.

Mortality due to causes other than cardiovascular while on apixaban and VKA, for the trial period, were based on data from the ARISTOTLE trial [11]. Beyond the duration of the trial period (1.8 years), age- and gender-adjusted mortality due to causes other than cardiovascular, was obtained from Statistics Netherlands [27]–[29]. In addition to the mortality due to causes other than cardiovascular, an increase in mortality rates associated with AF, strokes by severity level, MI and SE, was incorporated as in previously published Markov models [17], [18].

### Utilities

A utility score specific for patients with AF was applied to all patients in the baseline health state of the model (Table S2) [30]. Upon the occurrence of stroke, MI or SE, utility scores were adjusted to account for the level of utility for AF and comorbid thromboembolic event jointly [30], [31]. Utility decrements following the occurrence of a certain bleeding event were applied additively for a specific time interval [32]. Finally, utility decrements reflecting the use of VKA (warfarin), apixaban and ASA were applied [32] [33].

### Costs

Prices of apixaban, defined as price per defined daily dose (2×5 mg), VKAs and ASA (100 mg) were taken from the official Dutch price list (Z-index) (Table S3) [34]. Cost of VKA was estimated as a weighted average cost of acenocumarol and fenprocoumon based on their usage in the Netherlands (80%:20%, respectively) [35]. In addition to anticoagulants’ costs, routine care cost representing medical specialist fee was added to all treatment alternatives and cost due to INR testing was added to treatment with VKAs [24].

Acute care costs associated with clinical events (IS and HS with different levels of severity, other ICH, other MB and CRNM bleedings, SE, MI) were adopted from previous costing studies conducted in the Netherlands and updated to the year 2013 using the Dutch inflation index (Table S3) [6], [23], [36], [37]. Patients surviving acute stroke and MI were assigned with long-term maintenance costs [38].

### Sensitivity analyses

Univariate sensitivity analyses were conducted in order to inspect the effects of the uncertainty in key input parameters and assumptions on the uncertainty in the final cost-effectiveness outcome. Furthermore, a probabilistic sensitivity analysis (PSA) was performed in order to simultaneously incorporate the uncertainty around all parameters in the CE analysis. Key input parameters in the deterministic analysis that were assumed random variables in the PSA were: event rates, utilities and costs. A gamma distribution was assigned to event rates, a beta distribution to utilities and a log-normal distribution to cost estimates. Results from the PSA were plotted on a CE plane and transformed into CE-acceptability curves (CEACs).

Finally, in order to investigate the impact of different levels of INR control on the estimated ICER, as is the case in the various Dutch thrombotic centers, scenario analyses were conducted. Four different scenarios were investigated. Specifically, one scenario assumed patients were equally distributed across centers with different cTTR, similarly to the patient allocation in the ARISTOTLE trial. Other scenarios assumed the allocation of all patients to the one of specific cTTR range (i.e. cTTR<52.38%, 52.38%≤cTTR<66.02% and cTTR≥76.51%) that was different from the range in the base-case analysis (i.e. cTTR = 72.48%).

## Results

The number of events associated with the use of VKAs and apixaban in a cohort of 1,000 patients with non-valvular AF, as well as the costs related to those events and the anticoagulant treatment, are presented in Table 2. Specifically, the incremental difference in the number of events observed over a lifetime horizon in the apixaban treatment scenario compared to the VKA treatment scenario was: ten less stroke or SE events (including first and recurrent IS and HS), nine less other ICHs, 12 more other MBs, three less MIs and 58 less CRNM bleedings. A comparable number of other treatment discontinuations was observed in both apixaban and VKA treatment scenarios (648 and 652 respectively). Finally, treatment with apixaban was estimated to provide an additional 0.18 QALYs or 0.18 LYs compared to treatment with VKA over a lifetime horizon (Table 2).

**Table 2:** Stroke and other thromboembolic and bleeding complications and related costs within a hypothetical patient population of 1,000 subjects receiving apixaban and VKA over a lifetime horizon.

	Apixaban			VKA		
	Numberof events	Acute eventrelated lifetime costs p.p.	Long-term costs	Numberof events	Acute event related lifetime costs p.p.	Long-term costs
IS						
-Mild, non-fatal	96.32	€1,429	€638	93.01	€1,379	€614
-Moderate, non-fatal	83.62	€3,285	€2,612	89.30	€3,563	€2,921
-Severe, non-fatal	32.86	€1,586	€643	34.31	€1,675	€699
-Fatal	30.36	€67		29.05	€64	
Sum	243.15	€10,259		245.67	€10,915	
Recurrent IS						
-Mild, non-fatal	10.63	€149	€38	10.81	€152	€37
-Moderate, non-fatal	4.21	€161	€225	4.28	€165	€244
-Severe, non-fatal	1.61	€75	€49	1.63	€77	€52
-Fatal	3.61	€7		3.67	€8	
Sum	20.06	€705		20.40	€735	
HS						
-Mild, non-fatal	4.86	€79	€41	5.64	€93	€49
-Moderate, non-fatal	7.78	€339	€333	5.70	€247	€241
-Severe, non-fatal	4.47	€226	€105	5.67	€295	€147
-Fatal	10.88	€25		17.68	€42	
Sum	27.99	€1,147		34.69	€1,115	
Recurrent HS						
-Mild, non-fatal	0.29	€4	€1	0.29	€4	€1
-Moderate, non-fatal	0.40	€16	€18	0.40	€16	€16
-Severe, non-fatal	0.13	€6	€5	0.13	€6	€6
-Fatal	0.44	€1		0.44	€1	
Sum	1.25	€51		1.25	€51	
SE						
-Non-fatal	24.10	€86		24.60	€88	
-Fatal	2.50	€0		2.55	€0	
Sum	26.60	€86		27.14	€88	
Other ICH						
-Non-fatal	11.72	€176		19.20	€303	
-Fatal	1.75	€0		2.87	€0	
Sum	13.47	€176		22.07	€303	
Other MBs						
-Non-fatal GI bleedings	78.14	€306		69.49	€274	
-Non-fatal Non ICH or Non GI related MBs	126.23	€496		123.45	€490	
-Fatal	4.17	€0		3.94	€0	
Sum	208.54	€802		196.88	€764	
CRNM bleeding	314.94	€7		372.69	€9	
MI						
-Non-fatal	76.39	€283	€596	78.85	€295	€630
-Fatal	14.34			14.79		
Sum	90.73	€879		93.64	€925	
						
Other treatment discontinuation	647.58			652.08		
Cost of anticoagulants		€3,870			€365	
Cost of routine care		€2,120			€2,067	
Cost of INR monitoring		€102			€1,018	
Total costs		€20,205			€18,353	

VKA, vitamin K-antagonist; p.p., per patient; IS, ischemic stroke; HS, hemorrhagic stroke; SE, systemic embolism; ICH, intracranial hemorrhage; MB, major bleeding; GI, gastrointestinal; CRNM, clinically relevant non-major; MI, myocardial infarction; INR, international normalized ratio.

Costs associated with handling stroke and thromboembolic events were lower in the apixaban treatment scenario compared to the VKA treatment scenario (€14,113 vs. €14,904) (Table 2). However, the overall anticoagulant treatment costs including the drug acquisition costs, costs of routine care and INR monitoring were higher with apixaban compared VKA (€6,092 vs. €3,449) (Table 2). Accounting for all the aforementioned costs resulted in an additional cost of €1,852, associated with the use of apixaban compared to VKA.

Finally, the summarized lifetime health and economic consequences of applying apixaban compared to VKA in 70-year old patients in the Netherlands yielded a base-case ICER of €10,576 per QALY gained or €10,529 per LY gained (Table 3).

**Table 3:** Incremental costs, QALYs and ICER for patients with non-valvular AF receiving anticoagulation therapy.

Treatment	Costs (€)	QALYs	LYs	Δ Cost	Δ QALY	Δ LYs	ICER (€/QALY)	ICER (€/LYs)
VKA	€18,353	7.00	10.26	€1,852	0.18	0.18	€10,576	€10,529
Apixaban	€20,205	7.18	10.44					

QALY, quality adjusted life year; AF, atrial fibrillation; LY, life-year; ICER, incremental cost-effectiveness ratio; VKA, vitamin-K antagonist.

### Sensitivity analyses

**Figure 2:**
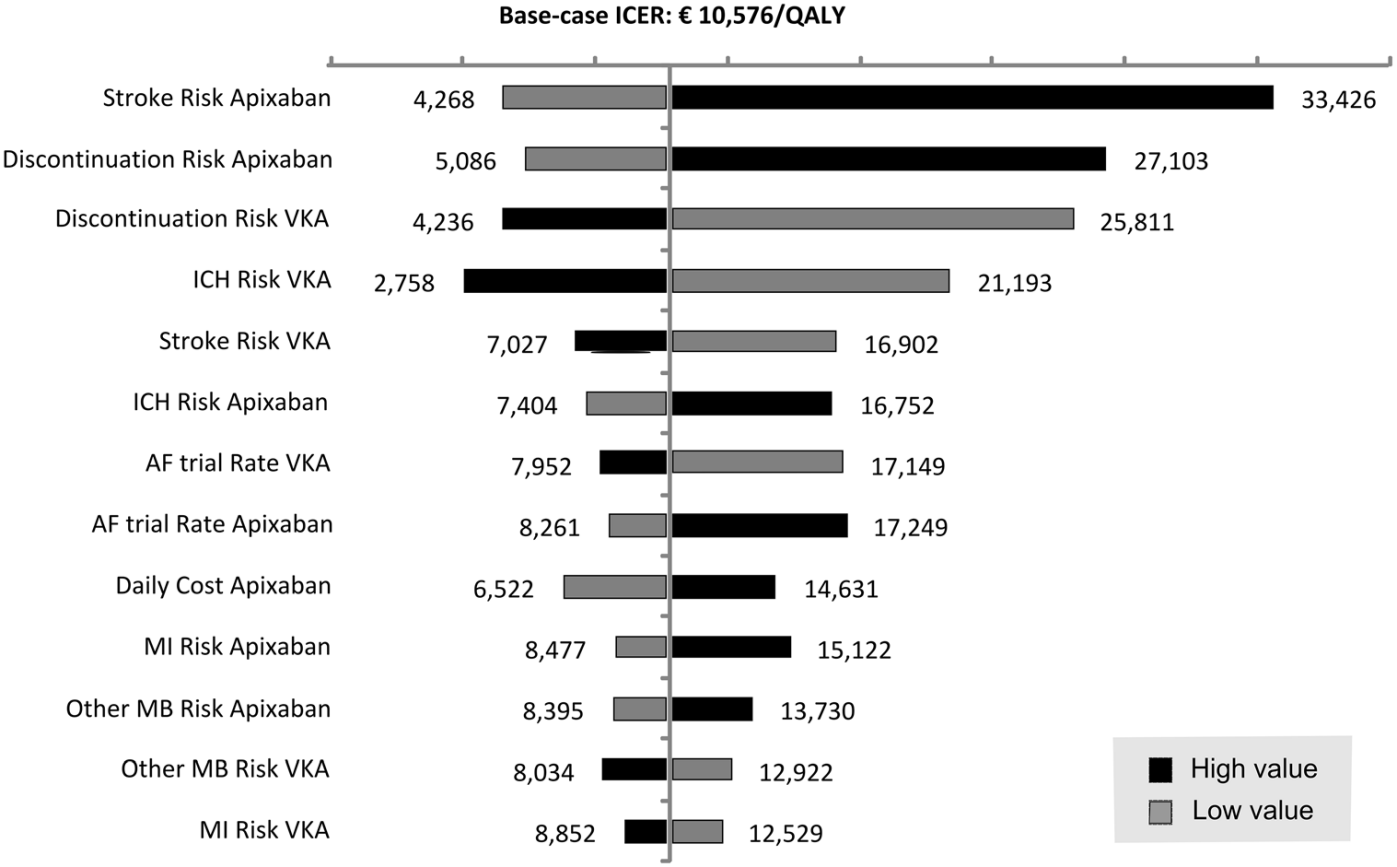
Tornado diagram illustrating results from sensitivity analyses for apixaban vs. vitamin-K antagonists. Black bars denote influence of the high value of the 95% confidence interval range and grey bars denote influence of the low value for parameters investigated. ICER, incremental cost-effectiveness ratio; QALY, quality adjusted life year; ICH, intracranial hemorrhage; AF, atrial fibrillation; MI, myocardial infarction; MB, major bleeding.

Figure 2 presents a tornado diagram illustrating the impact of varying each of key input parameters on the ICER while holding all the other model parameters fixed. The uncertainty around the absolute stroke risk under apixaban, the risks of treatment discontinuations under both apixaban and VKA and the risk of ICH under VKA, showed the highest impact on uncertainty in the estimated ICERs. In particular, when the absolute stroke risk or treatment discontinuations risk under apixaban would reach the upper limit of the 95% confidence interval (CI), ICERs would be €33,426 and €27,103 per QALY gained, respectively. At risks dropping to lower limits of 95% CIs, ICERs would fall to €4,268 or €5,086 per QALY gained, respectively. The uncertainty around the risks of treatment discontinuations under VKA led to comparable variation in estimated ICERs ranging from €4,236 to €25,811 per QALY gained.

**Figure 3:**
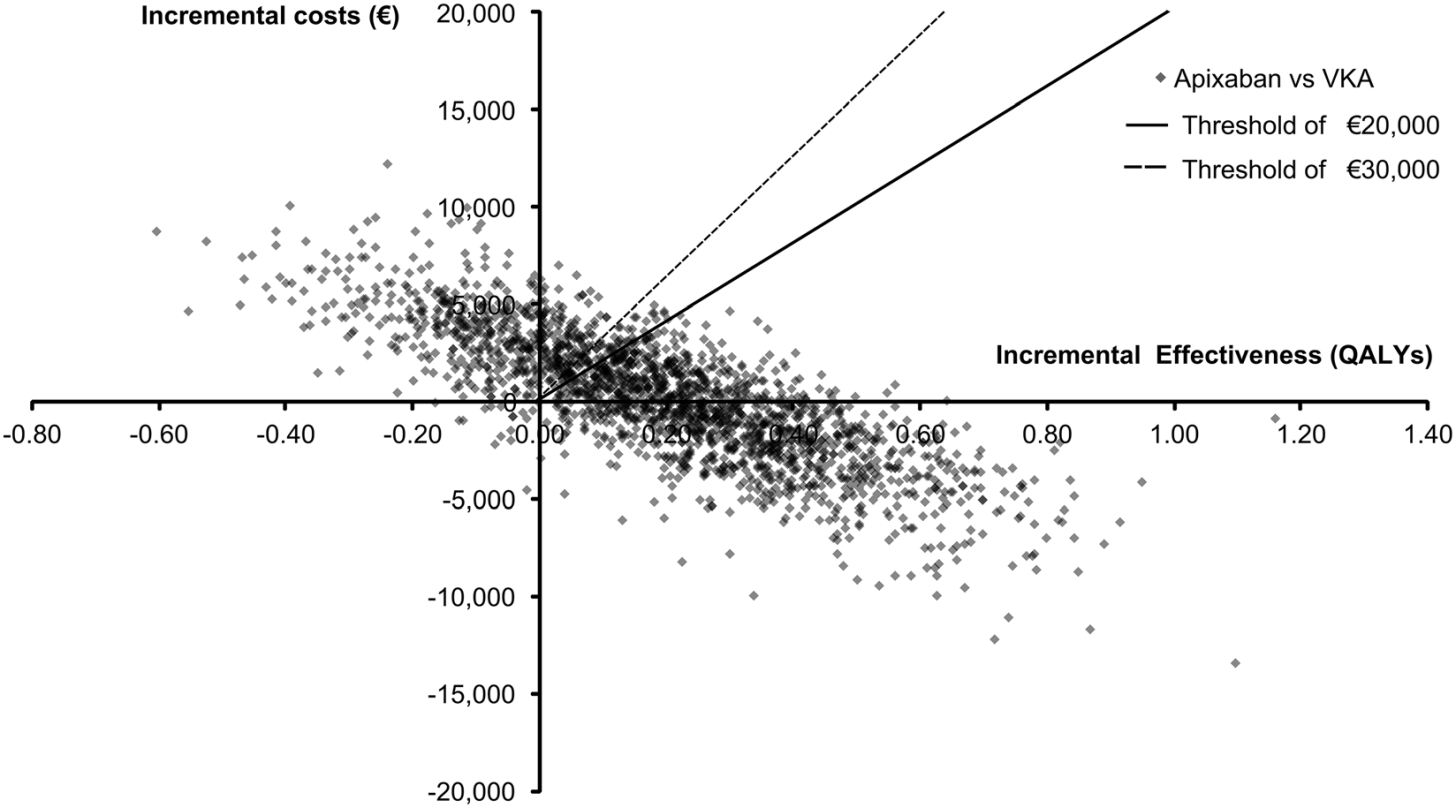
Incremental cost-effectiveness plane. Incremental cost-effectiveness plane presents the incremental cost-effectiveness ratios of apixaban compared to vitamin-K antagonists in patients with non-valvular atrial fibrillation, obtained through a probabilistic sensitivity analysis. Points below the diagonal dotted and the full line represent simulations in which apixaban was a cost-effective alternative at a threshold of €30,000/QALY and €20,000/QALY, respectively. QALY, quality adjusted life year; VKA, vitamin-K antagonists.

**Figure 4:**
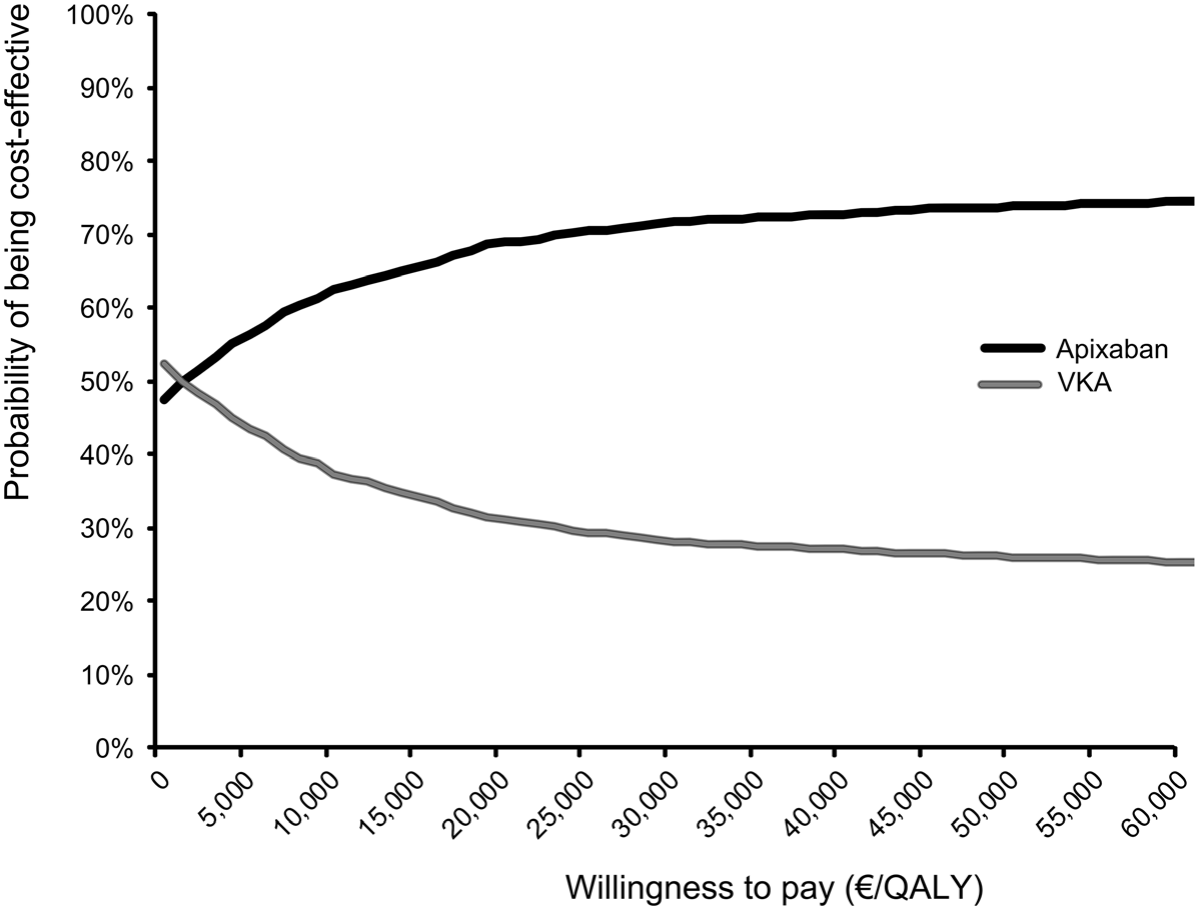
Cost-effectiveness acceptability curves for the treatment with apixaban and VKA in non-valvular atrial fibrillation. The cost-effectiveness acceptability curve assesses the probability that the estimated incremental cost-effectiveness ratio is under a certain willingness to pay threshold. VKA, vitamin-K antagonist; QALY, quality adjusted life year.

The results of 2,000 iterations in PSA are presented through an incremental CE plane in Figure 3. The ellipsoid shape of this incremental CE plane indicated a negative correlation between incremental costs and incremental effects. Transforming the results of a CE plane to CEACs shows that apixaban was cost-effective at alternative willingness to pay (WTP) thresholds of €20,000/QALY and €30,000/QALY in 68% and 72% of simulations respectively (Figure 4). Accordingly, VKA was estimated to be the preferred alternative over apixaban at the aforementioned WTP thresholds in 32% and 28% of simulations respectively.

The impact of different levels of INR control on the estimated ICER was explored through scenario analyses. Specifically, the level of INR control is applied for the estimation of the rates of IS, ICH, other MB and CRNM bleeding and therefore can have an indirect impact on the estimated ICER. Across the scenarios investigated, the majority of the aforementioned rates was estimated to be lower with apixaban compared to VKA. IS rate with apixaban was estimated to be higher than with VKA (i.e. 1.316 and 1.159, respectively) only in the scenario assuming allocation of patients in the range 52.38%≤cTTR<66.02%. Finally, the estimated ICER was in range from €27 to €12,662/QALY or from €22 to €12,905/LY across the scenarios explored (Table 4).

**Table 4:** Scenario analyses on the impact of different levels of INR monitoring.

Scenario	Treatment	IS rate	ICH rate	OtherMBs rate	CRNMbleeding rate	Costs (€)	QALYs	LYs	Δ Cost	Δ QALY	Δ LYs	ICER(€/QALY)	ICER(€/LY)
cTTR<52.38%	VKA	1.787	0.959	1.765	2.622	€20,328	6.81	10.00	€9	0.34	0.40	€27	€22
	Apixaban	1.213	0.292	0.999	1.271	€20,337	7.15	10.40					
52.38%≤cTTR<66.02%	VKA	1.159	0.912	2.093	2.657	€19,034	6.92	10.15	€2,039	0.16	0.15	€12,662	€12,905
	Apixaban	1.316	0.502	1.384	1.788	€21,074	7.08	10.30					
cTTR≥76.51%	VKA	0.831	0.708	2.857	3.364	€18,285	7.00	10.27	€1,261	0.24	0.26	€5,232	€4,698
	Apixaban	0.738	0.181	2.442	3.043	€19,546	7.24	10.53					
Equal distribution of patients across cTTRs	VKA	1.186	0.800	2.270	2.995	€19,013	6.93	10.17	€1,283	0.23	0.25	€5,599	€5,085
	Apixaban	1.044	0.330	1.790	2.083	€20,296	7.16	10.42					

Event rates adjusted for the level of INR monitoring, incremental costs, QALYs and ICERs.

INR, international normalized ratio; QALY, quality adjusted life year; ICER, incremental cost-effectiveness ratio; IS, ischemic stroke; ICH, intracranial hemorrhage; MB, major bleeding; CRNM, clinically relevant non-major; LY, life-year; cTTR, clinic time in therapeutic range; VKA, vitamin-K antagonist.

## Discussion

This economic evaluation estimated the CE of apixaban compared to VKA for prevention of stroke and other thromboembolic events in patients with non-valvular AF in the Netherlands. Apixaban was shown to be a cost-effective alternative to treatment with VKA with an ICER of €10,576/QALY or €10,529/LY. Notwithstanding that both the Dutch-specific level of INR monitoring (TTR = 72.48%) and a weighted level of baseline CHADS_2_ stroke risk were incorporated in this analysis, the specific long-term health and economic benefits of treatment with apixaban are evident. Those benefits correspond with a lower number of stroke and thromboembolic events as well as a generally better safety profile (i.e. less ICH and CRNM bleeding events) that is associated with the use of apixaban when compared to VKA. However, the number of other MBs was higher in the apixaban treatment scenario compared to VKA scenario. This finding can be explained by a relatively small difference in risks of MBs between the two comparators and a higher number of survivors in each model cycle that would be exposed to those risks in the apixaban treatment scenario.

Yet, the base-case ICER was found to be below the Dutch informal WTP threshold of €20,000/QALY in 68% of PSA simulations mainly reflecting the uncertainty in the apixaban absolute stroke risk and the risks of treatment discontinuations under both comparators. The major impact of uncertainty in those risks on both incremental effects and incremental costs was visualized in univariate sensitivity analyses’ tornado diagram (Figure 2) and PSA’s incremental CE plane (Figure 3). Specifically, the estimated ICERs in univariate and probabilistic sensitivity analyses, the specific shape of incremental CE plane and the overall number of simulated ICERs below a certain WTP threshold are mainly driven by the uncertainty in absolute stroke risk and treatment discontinuations risk. Notably, the relevance of the uncertainty in the apixaban absolute stroke risk can be directly attributed to its impact on the occurrence of stroke events and their related costs of treatment and reduced quality of life. In particular, the influence of the risk of treatment discontinuations can be indirectly explained by the choice of a second-line treatment that would follow after those discontinuations. In this analysis ASA was chosen to be a second-line treatment even though it provides less protection from various thromboembolic and bleeding events. Therefore, uncertainty in the risk of treatment discontinuations would in the case of a higher level of risk, lead to more patients being treated with ASA and consequently to a higher number of stroke and thromboembolic events.

Finally, the results of the scenario analyses examining the impact of different levels of INR control were quite robust, resulting in ICER estimates bellow €20,000/QALY in all the scenarios.

### Comparison with other studies

Findings of this analysis regarding the long-term health effects and economic consequences of using apixaban compared to VKA are similar to the results of other analyses studying apixaban in the US setting [39]–[41]. Harrington et al. evaluated the use of NOACs compared to warfarin and estimated an ICER of $15,026/QALY for apixaban [39]. Furthermore, the use of apixaban was reported to be a cost-effective alternative with $11,400/QALY compared to warfarin in the study of Kamel et al [40]. Finally, Lee et al. found apixaban to be a cost-saving option compared to warfarin [41]. Differences in the observed ICERs might be explained by different modelling assumptions and inputs that were used. Also differences in economic consequences associated with the use of anticoagulants could be attributed to the variability in country-specific cost estimates and the choice of study perspective (e.g. societal [40], [41]). Finally, differences in the underlying patients’ characteristics, modes of INR control and various modelling assumptions such as inclusion of multiple recurrent events [41] or different comorbid health states (e.g. transient ischemic attack [40]) could additionally hinder comparability between the study results.

### Strengths and limitations

Our study examines the potential use of apixaban for the prevention of stroke and other thromboembolic events in patients with non-valvular AF in the Dutch setting. Country-specific cost estimates, nation-specific background mortality and conjoint influence of the level of INR control and the allocation of baseline CHADS_2_ stroke risk in Dutch population on the events rates were implemented in this analysis. Furthermore, the impact of different levels of INR control was examined in this study. Comparable to similar CE studies, the health states of IS, SE, ICH, MI and CRNM bleedings were incorporated in this analysis. Finally, unlike other aforementioned analysis, utility estimates of stroke, MI and SE were estimated to reflect the level of utility for joint health states, using specific methodologies [42].

Our analysis has several limitations that may restrict the interpretation of results in a broader context. One limitation might be that the event rates incorporated in the decision model were assumed to be constant through life even though they were based on ARISTOTLE trial with an average follow-up of 1.8 years. This assumption, however, was partly corrected by applying event-specific rates that account for age-related increase in risk. In addition, multiple thromboembolic events were not incorporated in the decision model due to the lack of epidemiological evidence. Incorporating multiple thromboembolic events could nevertheless lead to a more favorable ICER which makes our analysis more conservative. A further potential limitation in our analysis is the assumption that a certain number of patients who experience ICH or other MB as well as all patients that discontinue treatment for reasons other than thromboembolic events, switch to a treatment with ASA. Noticeably, second-line treatment with ASA will provide a different level of protection from stroke and thromboembolic events when compared to initially assigned anticoagulants. In a real-life setting, patients might alternatively be switched to other NOACs or VKAs. However, current guidelines have no clear recommendations for the treatment following bleeding events or treatment discontinuations. Finally, our analysis does not reflect a societal perspective. In patients with non-valvular AF younger than 65 years, incorporating a societal perspective is of major importance given that this patient population encounters a significant productivity loss. Accounting for the productivity loss in the aforementioned patient population, would lead to even more favorable ICERs.

### Implications for practice and future research

Our findings showed apixaban to be a cost-effective alternative to VKAs in the Dutch setting with 68% of chances if a willingness-to-pay threshold is set to €20,000/QALY. Having in mind the high cost of illness due to AF and associated comorbid events, applying an effective and safe treatment in patients is of high importance. In the ARISTOTLE and AVERROES trials apixaban was shown to be a valuable alternative to VKAs and ASA regarding the effectiveness and safety issues. However “real life” long-term benefits of the use of apixaban as well as other NOACs still need to be proven, primarily regarding the patients’ adherence to them [43]. Additionally, the regulations regarding the choice of a second-line treatment in the case of adverse drug reactions need to be more clearly specified.

Apixaban is a NOAC which was recently approved for use in Europe, just after two other NOACs, rivaroxaban and dabigatran, were introduced. Further investigation should be directed to estimating comparative effectiveness and CE among the individual NOACs in the Dutch setting.

## Supporting Information

Table S1 **Rates of events while on apixaban, VKA and ASA used in estimating the transition probabilities in the model.** VKA, vitamin K-antagonist; ASA, acetylsalicylic acid; IS, ischemic stroke; HR, hazard ratio; cTTR, clinic time in therapeutic range; MI, myocardial infarction; ICH, intracranial hemorrhage; MB, major bleeding; CRNM, clinically relevant non-major; SE, systemic embolism.
(DOCX)

Table S2 **Utility parameters applied in the model.** AF, atrial fibrillation; SE, systemic embolism; MI, myocardial infarction; ICH, intracranial hemorrhage; MB, major bleeding; CRNM, clinically relevant non-major; VKA, vitamin K-antagonist; ASA, acetylsalicylic acid. *Utility estimates that were available only as single point estimates, were assumed to follow a beta distribution with a 10% standard deviation of the mean. ^§^Utilities were calculated based on the method for predicting utility for joint health states by Bo Hu [42].
(DOCX)

Table S3 **Cost parameters applied in the model.** VKA, vitamin K-antagonist; ASA, acetylsalicylic acid; SE, systemic embolism; ICH, intracranial hemorrhage; GI, gastrointestinal; CRNM, clinically relevant non-major; GP, general practitioner; MI, myocardial infarction. ^‡^Cost estimates that were available only as single point estimates, were assumed to follow a log-normal distribution with a coefficient of variation equal to 0.25. ^§^Cost of VKA was estimated as a weighted average cost of acenocumarol and fenprocoumon based on their usage in the Netherlands [34] ^¶^Stroke related costs were adjusted to fit the design of a decision model. Specifically, acute and long-term one-month maintenance costs were estimated. *Assumed to be equal to the cost of pulmonary embolism. ^#^Assumed to be the same as cost of acute mild stroke ^¥^Assumed to be the same as cost of GI bleeds.
(DOCX)
