# Supplementary material for: Economic Evaluation of Apixaban for the Prevention of Stroke in Non-Valvular Atrial Fibrillation in the Netherlands
Source: PLoS One. 2014 Aug 5;9(8):e103974. doi: 10.1371/journal.pone.0103974 (PMC4122386; doi:10.1371/journal.pone.0103974)
Supplement: Table S1 — Rates of events while on apixaban, VKA and ASA used in estimating the transition probabilities in the model. VKA, vitamin K-antagonist; ASA, acetylsalicylic acid; IS, ischemic stroke; HR, hazard ratio; cTTR, clinic time in therapeutic range; MI, myocardial infarction; ICH, intracranial hemorrhage; MB, major bleeding; CRNM, clinically relevant non-major; SE, systemic embolism. (DOCX) [file pone.0103974.s001.docx]

**Supplementary material**

**Table 1. Rates of events while on apixaban, VKA and ASA used in estimating the transition probabilities in the model.**

| Parameter | Apixaban | VKA | ASA | Reference |
| --- | --- | --- | --- | --- |
| IS rate by CHADS_2_ score |  |  |  |  |
| 1 | 0.52 | 0.46 |  | [11],[15],[17],[19] |
| 2 | 0.52 | 0.46 |  | [11],[15],[17],[19] |
| 3 | 0.95 | 0.93 |  | [11],[15],[17],[19] |
| 4 | 1.53 | 1.94 |  | [11],[15],[17],[19] |
| 5 | 1.53 | 1.94 |  | [11],[15],[17],[19] |
| 6 | 1.53 | 1.94 |  | [11],[15],[17],[19] |
| 7 | 1.53 | 1.94 |  | [11],[15],[17],[19] |
| IS HR by cTTR |  |  |  |  |
| cTTR < 52.38% | 0.92 | 1.54 |  | [11],[15],[17],[19] |
| 52.38% ≤ cTTR < 66.02% | 1.00 | 1.00 |  | [11],[15],[17],[19] |
| 66.02% ≤ cTTR < 76.51% | 0.69 | 0.84 |  | [11],[15],[17],[19] |
| cTTR ≥ 76.51% | 0.56 | 0.72 |  | [11],[15],[17],[19] |
| Rate of IS (per 100 patient years) |  |  | 3.45 | [11],[15],[17],[19] |
| Rate of ICH (per 100 patient years) | 0.33 | 0.80 | 0.32 | [11],[15],[17],[19] |
| ICH HR by cTTR |  |  |  |  |
| cTTR < 52.38% | 0.58 | 1.05 |  | [11],[15],[17],[19] |
| 52.38% ≤ cTTR < 66.02% | 1.00 | 1.00 |  | [11],[15],[17],[19] |
| 66.02% ≤ cTTR < 76.51% | 0.69 | 0.68 |  | [11],[15],[17],[19] |
| cTTR ≥ 76.51% | 0.36 | 0.78 |  | [11],[15],[17],[19] |
| Rate of other MBs (per 100 patient years) | 1.79 | 2.27 | 0.89 | [11],[15],[17],[19] |
| Other MBs HR by cTTR |  |  |  |  |
| cTTR < 52.38% | 0.72 | 0.84 |  | [11],[15],[17],[19] |
| 52.38% ≤ cTTR < 66.02% | 1.00 | 1.00 |  | [11],[15],[17],[19] |
| 66.02% ≤ cTTR < 76.51% | 1.69 | 1.13 |  | [11],[15],[17],[19] |
| cTTR ≥ 76.51% | 1.77 | 1.37 |  | [11],[15],[17],[19] |
| Rate of CRNM bleedings (per 100 patient years) | 2.08 | 2.99 | 2.94 | [11],[15],[17],[19] |
| CRNM bleedings HR by cTTR |  |  |  |  |
| cTTR < 52.38% | 0.71 | 0.99 |  | [11],[15],[17],[19] |
| 52.38% ≤ cTTR < 66.02% | 1.00 | 1.00 |  | [11],[15],[17],[19] |
| 66.02% ≤ cTTR < 76.51% | 1.25 | 1.26 |  | [11],[15],[17],[19] |
| cTTR ≥ 76.51% | 1.70 | 1.27 |  | [11],[15],[17],[19] |
| Rate of MI (per 100 patient years) | 0.53 | 0.61 | 1.11 | [11],[15],[17],[19] |
| Rate of other treatment discontinuations (unrelated to stroke and bleedings) (per 100 patient years) | 13.42 | 14.54 | 19.65 | [11],[15],[17],[19] |
| Rate of SE (per 100 patient years) | 0.09 | 0.10 | 0.4 | [11],[15],[17],[19] |
| Death rate during trial period (per 100 patient years) | 3.08 | 3.34 | 3.59 | [11],[15],[17],[19] |
| Background mortality after trial period | Age- and gender-adjusted non-CVD mortality | | | [27]-[29] |

VKA, vitamin K-antagonist; ASA, acetylsalicylic acid; IS, ischemic stroke; HR, hazard ratio; cTTR, clinic time in therapeutic range; MI, myocardial infarction; ICH, intracranial hemorrhage; MB, major bleeding; CRNM, clinically relevant non-major; SE, systemic embolism.
