# Supplementary material for: Economic Evaluation of Apixaban for the Prevention of Stroke in Non-Valvular Atrial Fibrillation in the Netherlands
Source: PLoS One. 2014 Aug 5;9(8):e103974. doi: 10.1371/journal.pone.0103974 (PMC4122386; doi:10.1371/journal.pone.0103974)
Supplement: Table S2 — Utility parameters applied in the model. AF, atrial fibrillation; SE, systemic embolism; MI, myocardial infarction; ICH, intracranial hemorrhage; MB, major bleeding; CRNM, clinically relevant non-major; VKA, vitamin K-antagonist; ASA, acetylsalicylic acid. *Utility estimates that were available only as single point estimates, were assumed to follow a beta distribution with a 10% standard deviation of the mean. §Utilities were calculated based on the method for predicting utility for joint health states by Bo Hu [42]. (DOCX) [file pone.0103974.s002.docx]

**Supplementary material**

**Table 2. Utility parameters applied in the model.**

| Parameter | Mean | Range* | Reference |
| --- | --- | --- | --- |
| Baseline Utility for AF | 0.6980 | 0.5532-0.8250 | [30] |
| Stroke mild | 0.6704 | 0.5330-0.7944 | [31] |
| Stroke moderate | 0.6165 | 0.4925-0.7333 | [31] |
| Stroke severe | 0.4416 | 0.3561-0.5289 | [31] |
| SE | 0.5769 | 0.4622-0.6876 | [30] |
| MI | 0.5328 | 0.4279-0.6363 | [30] |
| Disutility of other ICH (6 weeks) | 0.1385 | 0.1125-0.1667 | [32] |
| Disutility of other MBs (14 days) | 0.1385 | 0.1125-0.1667 | [32] |
| Disutility of CRNM bleedings (2 days) | 0.06 | 0.0488-0.0723 | [32] |
| Disutility of anticoagulation with VKA | 0.013 | 0.0106-0.0157 | [33] |
| Disutility of anticoagulation with ASA | 0.002 | 0.0016-0.0024 | [32] |
| Disutility of anticoagulation with apixaban | 0.002 | 0.0016-0.0024 | [32] |

AF, atrial fibrillation; SE, systemic embolism; MI, myocardial infarction; ICH, intracranial hemorrhage; MB, major bleeding; CRNM, clinically relevant non-major; VKA, vitamin K-antagonist; ASA, acetylsalicylic acid.

*Utility estimates that were available only as single point estimates, were assumed to follow a beta distribution with a 10% standard deviation of the mean.

^§^Utilities were calculated based on the method for predicting utility for joint health states by Bo Hu [42].
