# Supplementary material for: Economic Evaluation of Apixaban for the Prevention of Stroke in Non-Valvular Atrial Fibrillation in the Netherlands
Source: PLoS One. 2014 Aug 5;9(8):e103974. doi: 10.1371/journal.pone.0103974 (PMC4122386; doi:10.1371/journal.pone.0103974)
Supplement: Table S3 — Cost parameters applied in the model. VKA, vitamin K-antagonist; ASA, acetylsalicylic acid; SE, systemic embolism; ICH, intracranial hemorrhage; GI, gastrointestinal; CRNM, clinically relevant non-major; GP, general practitioner; MI, myocardial infarction. ‡Cost estimates that were available only as single point estimates, were assumed to follow a log-normal distribution with a coefficient of variation equal to 0.25. §Cost of VKA was estimated as a weighted average cost of acenocumarol and fenprocoumon based on their usage in the Netherlands [34] ¶Stroke related costs were adjusted to fit the design of a decision model. Specifically, acute and long-term one-month maintenance costs were estimated. *Assumed to be equal to the cost of pulmonary embolism. #Assumed to be the same as cost of acute mild stroke ¥Assumed to be the same as cost of GI bleeds. (DOCX) [file pone.0103974.s003.docx]

**Supplementary material**

**Table 3. Cost parameters applied in the model.**

| Parameter | Mean | Range^‡^ | Reference |
| --- | --- | --- | --- |
| Apixaban (daily) | € 2.28 | Fixed | [34] |
| VKA (daily)^§^ | € 0.03 | Fixed | [34] |
| ASA (daily) | € 0.15 | Fixed | [34] |
| Monitoring visit (per year) | € 224 | 163-308 | [34] |
| Routine care (per visit) | € 78.97 | 62-117 | [24] |
| Stroke^¶^ |  |  |  |
| Mild 0-6 months | € 16,097 | 11,712-22,124 | [6] |
| Mild 7-12 months | € 4,470 | 3,252-6,144 | [6] |
| Mild 13 -19 months men | € 1,174 | 854-1,614 | [6] |
| Mild 13 -19 months women | € 1,174 | 854-1,614 | [6] |
| Moderate 0-6 months | € 44,640 | 32,479-61,354 | [6] |
| Moderate 7-12 months | € 21,146 | 15,385-29,063 | [6] |
| Moderate 13 -19 months men | € 7,115 | 5,177-9,779 | [6] |
| Moderate 13 -19 months women | € 11,745 | 8,545-16,142 | [6] |
| Severe 0-6 months | € 54,678 | 39,783-75,150 | [6] |
| Severe 7-12 months | € 26,711 | 19,43-36,712 | [6] |
| Severe 13 -19 months men | € 9,055 | 6,588-12,445 | [6] |
| Severe 13 -19 months women | € 15,069 | 10,964-20,711 | [6] |
| Fatal stroke | € 2,988 | 2,876-3,102 | [38] |
| SE acute care (per episode)***** | € 4,995 | 3,634-6,865 | [37] |
| Other ICH^#^ | € 20,326 | 14,789-27,937 | Assumption |
| GI bleeds | € 4,995 | 3,635-6,866 | [37] |
| Non ICH and non-GI bleeds^¥^ | € 4,995 | 3,635-6,866 | Assumption |
| CRNM bleeds (assume a visit to GP) | € 30.71 | 22-42 | [24] |
| MI acute care (per episode) | € 5,021 | 4,936-5,106 | [36] |
| MI maintenance (per month) | € 196 | 183-206 | [38] |

VKA, vitamin K-antagonist; ASA, acetylsalicylic acid; SE, systemic embolism; ICH, intracranial hemorrhage; GI, gastrointestinal; CRNM, clinically relevant non-major; GP, general practitioner; MI, myocardial infarction.

^‡^Cost estimates that were available only as single point estimates, were assumed to follow a log-normal distribution with a coefficient of variation equal to 0.25.

^§^Cost of VKA was estimated as a weighted average cost of acenocumarol and fenprocoumon based on their usage in the Netherlands [34]

^¶^ Stroke related costs were adjusted to fit the design of a decision model. Specifically, acute and long-term one-month maintenance costs were estimated.

* Assumed to be equal to the cost of pulmonary embolism.

^#^Assumed to be the same as cost of acute mild stroke

^¥^Assumed to be the same as cost of GI bleeds
